# Confinement Effects on Carbon Dioxide Methanation: A Novel Mechanism for Abiotic Methane Formation

**DOI:** 10.1038/s41598-017-09445-1

**Published:** 2017-08-21

**Authors:** Thu Le, Alberto Striolo, C. Heath Turner, David R. Cole

**Affiliations:** 1 Department of Chemical Engineering, University College London, London, WC1E 6BT United Kingdom; 2 Department of Chemical and Biological Engineering, University of Alabama, Tuscaloosa, AL 35487 United States; 3 School of Earth Sciences, The Ohio State University, Columbus, Ohio, 43210 United States

## Abstract

An important scientific debate focuses on the possibility of abiotic synthesis of hydrocarbons during oceanic crust-seawater interactions. While on-site measurements near hydrothermal vents support this possibility, laboratory studies have provided data that are in some cases contradictory. At conditions relevant for sub-surface environments it has been shown that classic thermodynamics favour the production of CO_2_ from CH_4_, while abiotic methane synthesis would require the opposite. However, confinement effects are known to alter reaction equilibria. This report shows that indeed thermodynamic equilibrium can be shifted towards methane production, suggesting that thermal hydrocarbon synthesis near hydrothermal vents and deeper in the magma-hydrothermal system is possible. We report reactive ensemble Monte Carlo simulations for the CO_2_ methanation reaction. We compare the predicted equilibrium composition in the bulk gaseous phase to that expected in the presence of confinement. In the bulk phase we obtain excellent agreement with classic thermodynamic expectations. When the reactants can exchange between bulk and a confined phase our results show strong dependency of the reaction equilibrium conversions, ${{\text{X}}}_{{\bf{C}}{{\bf{O}}}_{{\bf{2}}}}$, on nanopore size, nanopore chemistry, and nanopore morphology. Some physical conditions that could shift significantly the equilibrium composition of the reactive system with respect to bulk observations are discussed.

## Introduction

The discovery of submarine hydrothermal vent systems some forty years ago prompted a whole new field of exciting multi-disciplinary research, which has reshaped the core hypothesises of the origin of life^1, 2^. The high concentrations of organic compounds, including methane, measured directly at the vents (black smokers) suggest the abiotic synthesis of organic molecules, which could have implications for the origin and sustenance of life on early Earth^3^. It is within this context that the possibility of abiotic hydrocarbon production has been extensively tested at hydrothermal conditions. As the industrial Fischer-Tropsch process involves hydrocarbons production by CO hydrogenation, it has become common to refer to those possible chemical routes to model abiotic organic formation from various carbon sources as Fisher-Tropsch type (FTT) reactions^3, 4^. Within this general FTT formalism, aqueous CO_2_ could be reduced by H_2_:

1 $$C{O}_{2}+[(2+m/2n){H}_{2}]\to (1/n){C}_{n}{H}_{m}+2{H}_{2}O$$

High hydrogen concentrations could result from water-ferrous silicate interactions, known as serpentinization processes^5^, including, e.g., the serpentinization of olivine, the predominant mineral of ultramafic rocks:

2 $$\mathop{6[(M{g}_{1.5}F{e}_{o.5})Si{O}_{4}]}\limits_{{\rm{olivine}}}+7{H}_{2}O\to \mathop{3[M{g}_{3}S{i}_{2}{O}_{5}{(OH)}_{4}]}\limits_{{\rm{serpentine}}}+\mathop{F{e}_{3}{O}_{4}}\limits_{{\rm{magnetite}}}+{H}_{2}$$

Serpentinization reactions typically occur at 300–500 °C, and are highly exothermic^6, 7^ [~660 MJ of thermal energy are released when 300 litres of water alter 1 cubic meter of rock via serpentinization^7^]. In the absence of heat dissipation mechanisms, this heat could raise the local rock temperature by ~260 °C^7^. In addition, as the serpentinization reaction proceeds the abundance of pores of size ~1–10 nm (i.e., nanopores) increases, as shown by neutron scattering experiments on samples obtained from the ca. 2 Ma Atlantis Massif oceanic core complex^8^. For completeness, it should be reported that serpentinization reactions can occur below 300 °C in submarine vents^9, 10^, and even below 100 °C in terrestrial ultramafic settings^11–13^.

There have been a number of recent hydrothermal experimental studies^14–17^ designed to test the hypothesis that CH_4_ is formed from serpentinization involving olivine reacted with various fluid types (e.g., H_2_O, H_2_O plus dissolved carbonate, seawater) at temperatures from 200 to 400 °C. In general, only small amounts of methane are generated, despite Reaction (1) being strongly favored by thermodynamics at low temperature and high reactant concentrations and long reaction times, i.e., 1000–2000 hours^6, 14^, which indicates large kinetic barriers. This is in contrast to select field studies of actively serpentinizing systems that can exhibit highly enriched CH_4_ in the bulk fluids^7, 10, 18–20^. Samples from the submarine hydrothermal vent systems represent the integrated signal of fluids (and gases) derived from a complex fracture and pore network feeding the vents. Reduction of CO_2_ degassed from magmatic reservoirs may be an additional source of carbon added to oceanic crust during the hydrothermal alteration process besides the dissolved carbon sourced from circulating seawater^21^. What remains uncertain is the distribution of oxidized and reduced forms of volatile carbon in the vent plumbing system and what role pores and micro-fractures play in the reactive-transport processes. To understand whether abiotic synthesis is possible, one would need to characterize the fluid composition in the pores, which is not technically achievable at present. Thus the goal of the present work, in which we employ reactive ensemble Monte Carlo molecular simulations,﻿ is to quantify the potential effect of pore confinement on the equilibrium conversion expected for CO_2_ methanation (i.e., conversion to CH_4_).

### Nanopore-Controlled Reactions

Confinement is known to alter thermodynamic and transport properties of fluids^22^. For example, we previously documented, using molecular dynamics (MD) simulations, the adsorption, structure, and diffusion of water^23–25^, aqueous electrolytes^26, 27^, pure hydrocarbons^28^, and mixtures of hydrocarbons and other substances^29–32^ in slit-shaped pores at various conditions of temperature, pressure and surface features. Our results showed, e.g., that preferential adsorption of CO_2_ to the pore surface can compete with the adsorption of hydrocarbons, lower the activation energy for diffusion, and enhance hydrocarbon mobility. Similarly, water can preferentially adsorb on silica-based pore surfaces. Others showed that confinement in nanopores can reduce the activation energy of various chemical reactions^33^, increase the reaction rate^34^, and enhance reaction yields^35–38^.

**Figure 1:**
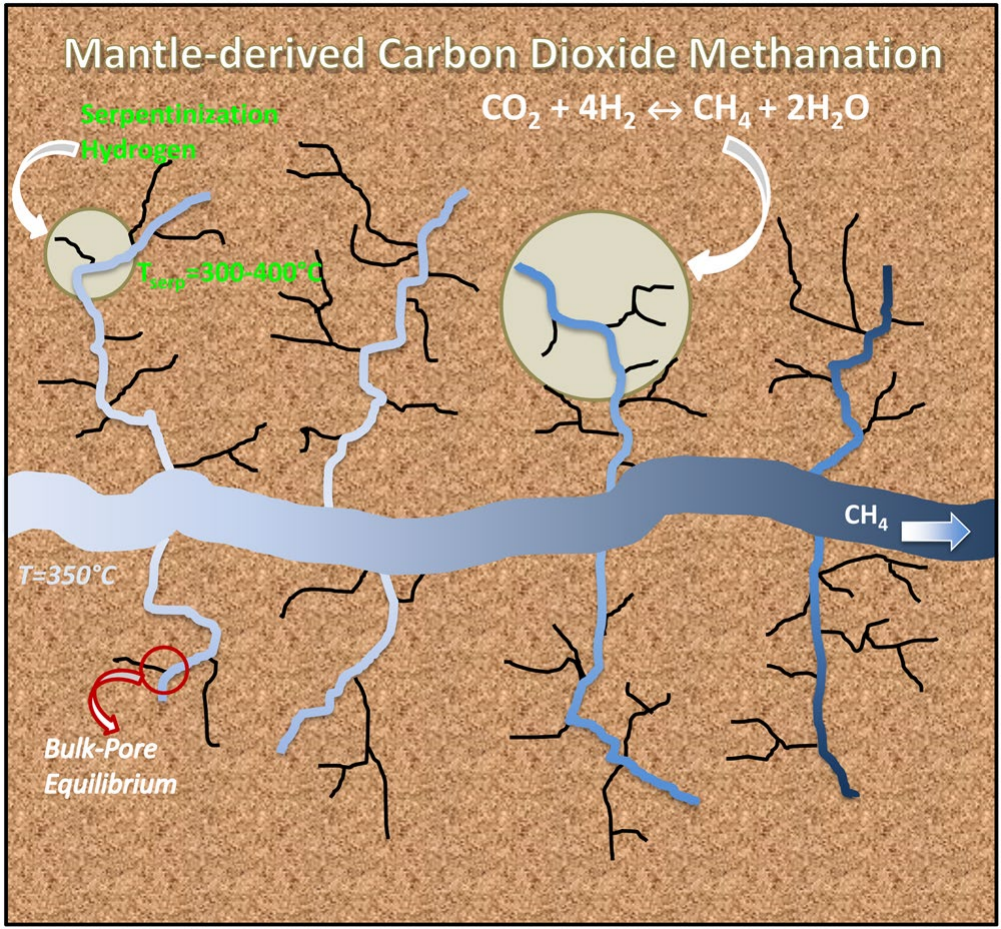
Schematic for the possible carbon dioxide methanation process considered in this work. Within oceanic crust, mantle-derived melt is intruded at shallow depths, heats the ultramafic ocean crust driving seawater circulation along natural fractures and microfractures, where serpentinization reactions take place. The H_2_ produced could then come in contact with carbon dioxide derived from deeply circulating seawater or the mantle.

Building on prior results, in this communication we explore whether the preferential adsorption of either CO_2_ or H_2_O on the pore surface can affect the equilibrium conversion of CO_2_ to CH_4_. The system we consider is meant to represent a nanoporous matrix in contact with a larger micro-fracture. Water, hydrogen, carbon dioxide and methane are free to exchange between the nanopores and the micro-fracture and to react within the various environments. The micro-fracture is large enough to provide a ‘bulk’ system. In Fig. 1 we present a schematic of the hypothetical scenario explored in this work, consistent with the conceptual model of a hydrothermal vent discussed by Ingebritsen *et al*.^39^. Our hypothesis is supported by recent evidence of the presence of nanoscale pore systems developed in serpentinites examined from the Atlantis Massif and the Duluth Complex^8^. The focus of this manuscript is to investigate whether it is possible that nanopore or nanofracture confinement shifts the equilibrium composition of CO_2_ methanation reactions. Because of computing-power limitations, only one nanopore is simulated explicitly. The molecules can exchange with the bulk (i.e., the microfracture) within our formalism.

While the interest on the effect of confinement on chemical reactions spans multiple disciplines, experimental measurements are highly challenging because of the difficulty encountered in directly monitoring fluid behavior in the confined spaces. This is particularly true for high temperature and high pressure conditions relevant to magma-hydrothermal systems. Theoretical approaches provide the opportunity to quantitatively probe the nanoconfined regime. Methods include variational perturbation theory^40^, integral equations^41–43^, and molecular simulations, which can provide electron- and/or atomistic-level insights. By implementing ab initio^44^ and force-field methods^45–47^ it is possible to study breaking and formation of chemical bonds. In this study we adopted the reaction ensemble Monte Carlo (RxMC) approach^48–50^, whose underlying concept is that equilibrium chemical conversion depends solely on thermodynamics. The RxMC method has been widely and successfully used to investigate bulk-phase reactions^49, 51^, reactions at high temperature^52, 53^ and high pressure^54, 55^, nanoconfined reactions^35, 37, 56^, and even reactions at interfaces^36, 57, 58^.

Among the family of FTT reactions summarised by Reaction (1), we consider here the methanation of carbon dioxide via the Sabatier reaction^59, 60^. Our technique does not allow us to investigate CO_2_ methanation that could occur microbially by methanogenesis^61^. The Sabatier reaction is expressed as:

3 $$C{O}_{2}+4{H}_{2}\leftrightarrow C{H}_{4}+2{H}_{2}O$$

The abiotic reduction of carbon dioxide to methane is thermodynamically favourable at low temperature, high pressure, and high hydrogen fugacity. The reaction is reversible and highly exothermic (Δ*H* = −165 kJ/mol CO_2_ at 25 °C and 1.01 bar)^62^. However, the double carbon-oxygen bonds of the stable CO_2_ molecule induce large kinetic barriers that in some cases prevent the reaction from reaching its expected conversion^63^. The use of catalysts is often essential to carry out the reaction, especially at low temperatures.

Park and McFarland proposed the CO_2_ methanation as a series of reactions^64^:

4 $$C{O}_{2}+{H}_{2}+3{H}_{2}\leftrightarrow {H}_{2}O+CO+3{H}_{2}\leftrightarrow {H}_{2}O+C{H}_{4}+{H}_{2}O$$

Gao *et al*. investigated the equilibrium bulk product fraction of Reaction (4) at 1.01 bar in the *T* range 200–800 °C using the Gibbs free energy minimization method^65^. The results show that from 200 to ~300 °C, little CO is present in the system and the conversion to CH_4_ is ~100%. From ~300 to ~500 °C, the amount of CO remains negligible; however, because CO_2_ methanation is strongly exothermic, the production of CH_4_ is hindered in this *T* range.

While the majority of existing experimental and/or theoretical studies dedicated to the above reactions for industrial uses focus on optimizing the catalysts, we are concerned here only with the equilibrium conversion. As such, the choice of catalyst, the transient states and kinetics effects are not considered. For the sake of simplicity, in this work we focus on the direct methanation of CO_2_ (no CO intermediate), Reaction (3), at moderate pressures 10–50 bar in the range of temperatures 200–700 °C. The pressure-temperature conditions chosen for the present study provide ideal scenarios for proof-of-principle studies: at low temperatures and high pressures the equilibrium conversion of Reaction (3) is expected to favour CH_4_ production even in bulk systems, hence at such conditions confinement is likely to have little effect. Although temperatures 400 °C and above are more representative of deeper ocean crustal conditions than of hydrothermal vents, where exit temperatures tend to not exceed ∼350 °C, it should be pointed out that the Brandon, the 5 S MAR, and the Piccard field sites show seafloor temperatures near 400 °C^66–68^, and that in other vents higher temperatures are reached before venting (e.g., in the Lucky Strike vent)^69^. The model considered here is consistent with the conceptual model of a hydrothermal vent discussed by Ingebritsen *et al*.^39^ and Sleep *et al*.^61^. As mentioned above, the serpentinization reaction is highly exothermic, and could lead to local temperature increases. High temperatures and moderate pressures are chosen here because they facilitate the completion of the calculations, compared to moderate temperatures and high pressures, which would be more realistic. However, this choice of conditions is not expected to affect the proof of principle nature of this work because high temperatures promote desorption of fluids from nanopores, reducing the effect of confinement on the results, and because high pressures are expected to shift the equilibrium composition of Reaction (3) towards the production of CH_4_. Hence considering pressures at least one order of magnitude lower than those expected in realistic scenarios allows us to test more reliably the effect of confinement on the equilibrium conversion of Reaction (3).

To test the hypothesis that nanoconfinement impacts the equilibrium conversion of Reaction (3), we selected a simple pore chemistry – SiO_2_. While the SiO_2_ pore model (dominated by SiO_4_ tetrahedra) does not exactly represent the mineralogy considered in the serpentinization Reaction (2), the cristobalite crystal surface with fully protonated non-bridging oxygen atoms can be considered a reasonable proxy for hydrophilic silicate pore surfaces^70^. The silica surfaces used in this work were obtained by cutting the β-cristobalite SiO_2_ crystal along the (1 1 1) crystallographic face.

This study stems from the hypothesis that preferential pore-fluid interactions affect the equilibrium conversion of Reaction (3). To alter pore-fluid interactions one could change the pore width, with smaller pores in general attracting fluid molecules more strongly than wider pores. The pore width is set at 2 nm, which is expected to strongly attract fluid molecules. Alternatively, pore-fluid interactions can be manipulated by changing the pore surface features, e.g., by altering the degree of protonation of the pore surfaces. Some of these manipulations are discussed in the Results section (and Supplementary Information). While this manuscript does not explore catalytic effects, it should be noted that some olivines contain Ni, Co or Cr, that could act as catalysts to increase the rate of reaction considered here^71^.

## Results and Discussion

### Bulk Phase

Gao *et al*.^65^ provide extensive information about the equilibrium constant *K* and product fractions as functions of temperature, pressure and CO_2_:H_2_ input ratio. The results show that, in summary, low *T*, high *P* and high H_2_ concentration favour CO_2_ methanation. To validate our RxMC simulations, we compare our simulated results to the data presented by Gao *et al*. In Supplemental Information we report the equilibrium constant reported by Gao *et al*. and that obtained using our method at *P* = 1.01 bar and CO_2_:H_2_ input ratio 1:4. The agreement is excellent. In Supplemental Information we also report the carbon dioxide conversion and mole percentage of all compounds as functions of temperature for the system just described, in the bulk phase. Despite a few small differences due to the choice of the simulated systems (see Supplementary Information for details, Figures S1, S2), our results suggest that the RxMC approach yields the expected conversions when implemented in the bulk.

**Figure 2:**
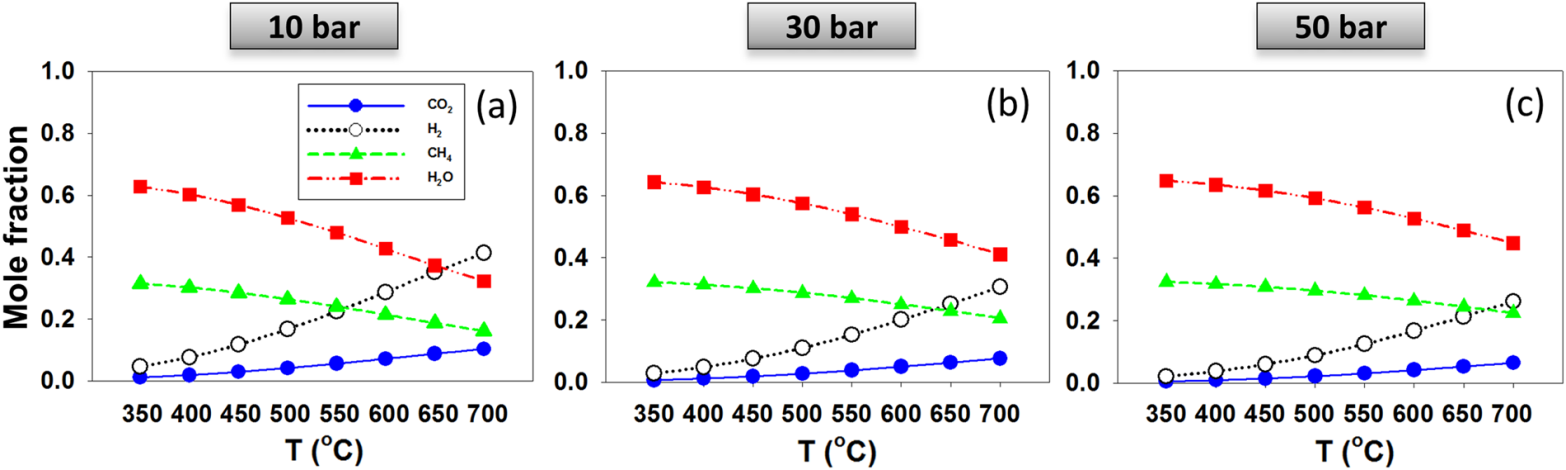
Simulated composition for the CO_2_ methanation reaction in the bulk phase at (**a**) 10 bar, (**b**) 30 bar, and (**c**) 50 bar. In all cases the initial CO_2_:H_2_ mole ratio was 1:4. In all figures, symbols are larger than statistical uncertainty.

To approximate conditions such as those found in deeper oceanic crust below hydrothermal vent environments or in subducting slabs of oceanic crust, we apply the RxMC formalism to analyse the equilibrium composition of the reactive system in the temperature range 350–700 °C at three pressures (10, 30 or 50 bar). While much higher pressures should be considered to emulate systems of oceanic crust relevance, our simulations become problematic at such conditions because the acceptance probability of attempted Monte Carlo moves decreases. However, increasing the pressure is expected to further shift the composition of Reaction (3) towards the formation of methane, as the number of moles decreases with the formation of products (i.e., 5 moles of reactants (one mole of CO_2_ and 4 moles of H_2_) yield 3 moles of products (one mole of CH_4_ and 2 moles of water)). The lower temperatures considered here are consistent with those expected in hydrothermal vent plumbing system, and the higher ones are expected for the fracture-pore network system associated with the deeper magma-hydrothermal system. The results, shown in Fig. 2, confirm that as *P* increases and *T* decreases the CH_4_ mole fraction at equilibrium increases. These observations are useful for comparing the results obtained when the reaction occurs in a nanoconfined system, which are discussed below.

### System in the Presence of NanoConfinement

#### Effects of Temperature and Pressure

We now consider our simulated results when Reaction (3) occurs where the bulk phase is at contact with one narrow pore. Bulk and pore fluids are in equilibrium with each other, and as such the fluid molecules can exchange between the two environments. The reaction can occur in either the bulk phase or within the pore. The choice of system conditions has been justified above. The system parameters are a compromise between computational feasibility, realistic description of the deeper crustal conditions, and the goal of assessing the effect of confinement on the equilibrium transformation of Reaction (3). The simulations are conducted at moderate temperatures (350–700 °C) and pressures (10, 30 or 50 bar). At these conditions the reaction in the bulk is far from completion (i.e., CO_2_ conversion to methane is less than 100%, as shown in Fig. 2) and the fluid species can adsorb in the pore. Under these circumstances it is expected that confinement can strongly affect the equilibrium conversion. In all simulations, the initial CO_2_:H_2_ ratio was kept 1:4. These highly reducing compositions are chosen because they provide abundance of hydrogen, allowing us to study the effect of confinement on the equilibrium composition. Experimental quantification of the composition of dissolved gases in representative hydrothermal vents suggests that the CO_2_:H_2_ ratio can be as high as 10 or more, but in the case of the Lost City vent the ratio can be lower than 1^4^. Because of the proof-of-principle nature of this work, we consider the initial molar ratio at 1:4 in most of the manuscript. Of course, increasing the amount of hydrogen shifts the reaction towards the production of methane.

**Figure 3:**
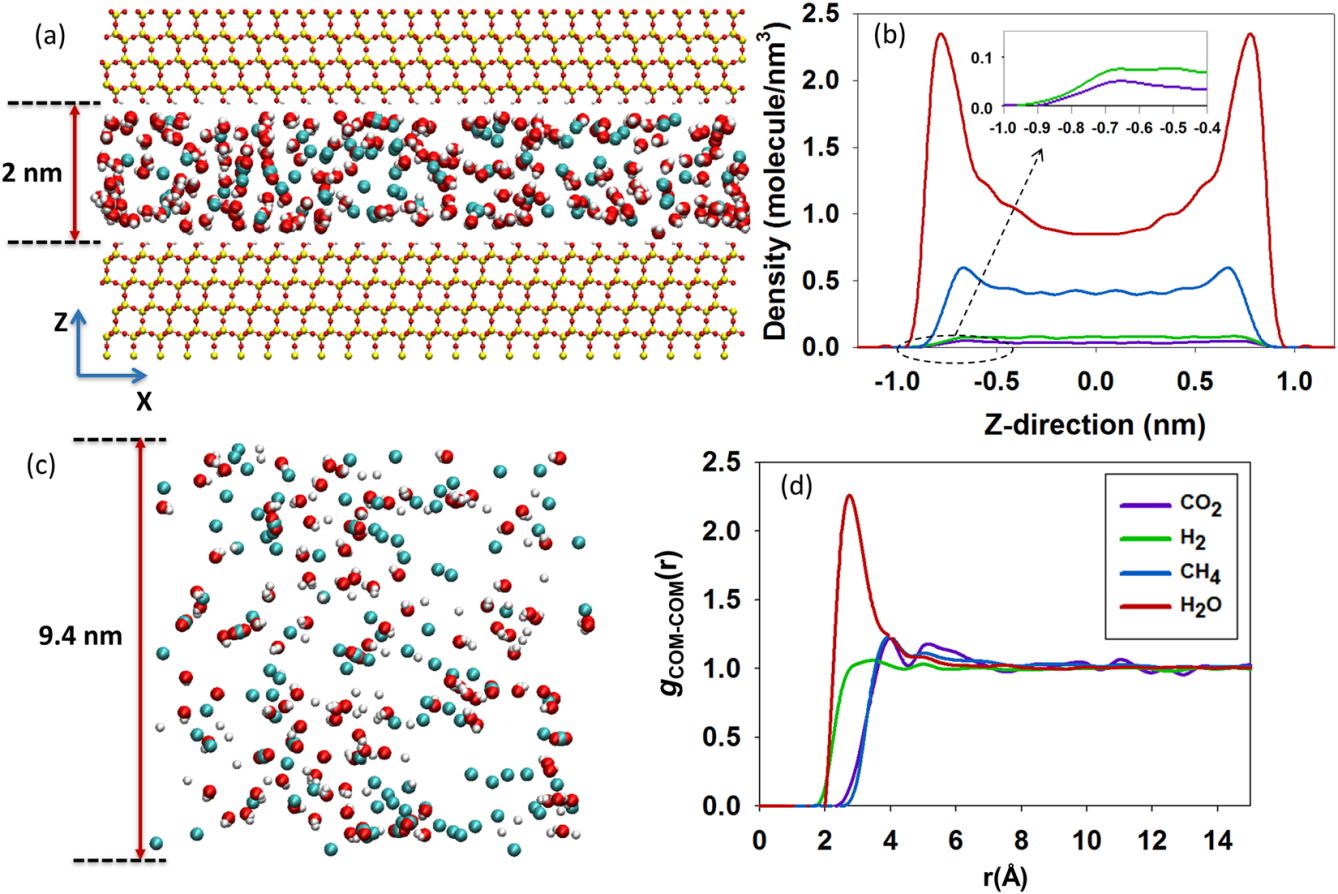
(**a**) Snapshot representing a simulation box containing the 2 nm silica pore at 650 °C and 50 bar. The solid silica slabs are continuous along X and Y directions. Cyan spheres are either CH_4_ or C in carbon dioxide, red is O, white is either H or H_2_, and yellow is Si. (**b**) Density profiles of CO_2_, H_2_, CH_4_ and H_2_O adsorbed within the *pore*. (**c**) Snapshot of the cubic bulk simulation box. (**d**) Radial distribution functions between the centres of mass ${g}_{COM-COM}(r)$ for the fluids in the *bulk phase*.

In Fig. 3 we report some characterization data obtained for a system of initial composition of 200 CO_2_ and 800 H_2_ that was allowed to react and reach equilibrium at 650 °C and 50 bar. In panel (a) we represent a sample snapshot for the slit-pore containing 6 CO_2_, 10 H_2_, 84 CH_4_ and 216 H_2_O molecules within the pore volume in equilibrium with a bulk phase (panel c) containing 12 CO_2_, 62 H_2_, 98 CH_4_ and 103 H_2_O molecules at 650 °C and 50 bar. The bulk region is a cubic box whose size can change. A typical volume at the end of a simulation run is of size 9.4 × 9.4 × 9.4 nm^3^, simulated within periodic boundary conditions in the 3 directions. We discuss below how the equilibrium composition of the system can depend on the relative amount of pore versus bulk volume.

Panel (b) of Fig. 3 reproduces the atomic density profiles of molecular center of mass (COMs) as a function of the distance Z perpendicular to the pore surface for the system depicted in panel (a). The reference Z = 0 is the centre of the pore. Statistically, only negligible amounts of CO_2_ and H_2_ adsorb into the pore. In the density profiles obtained for methane and water two distinct peaks are observed, symmetric with respect to the pore centre, and near the pore surfaces. The positions of the water peaks are closer to the pore walls, in comparison to the methane peaks. This confirms that water is strongly adsorbed on the pore surfaces, because of its capability to form hydrogen bonds with the hydroxyl groups on the pore walls. Although CO_2_ can also form hydrogen bonds with the surface –OH groups^72, 73^, there is too little H_2_ and CO_2_ available in the pore at reaction equilibrium, in comparison to methane and water, to observe significant peaks. Panel (d) of Fig. 3 shows the COM-COM radial distribution functions (RDFs) of the substances in the bulk phase. Only one distinct peak is observed in each RDF, indicating well-mixed gaseous structures. Note that both simulation snapshots confirm that the various components yield a well-mixed single-phase system, probably consistent with the supercritical conditions expected in sub-surface systems.

**Figure 4:**
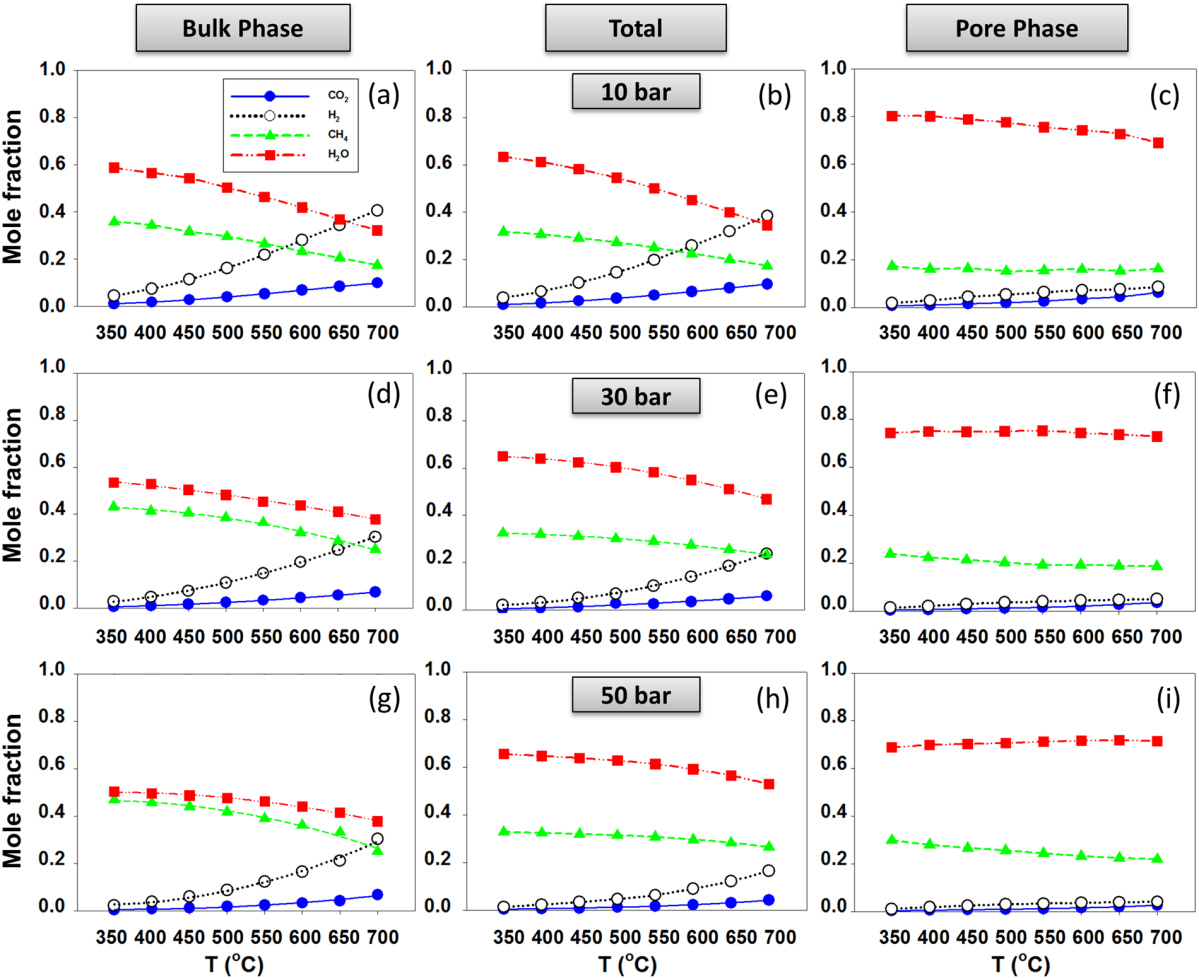
Simulated product fraction of CO_2_ methanation for the bulk phase (left panels) and pore phase (right panels) in equilibrium with each other at *T* and *P*. The middle panels show the total molecular fraction.

Figure 4 shows the mole fractions for each component at equilibrium. We differentiate the overall mole fraction (middle panels), from the mole fraction in the bulk (left panels) and in the pore (right panels). The overall data are obtained by considering both the fluids in the bulk and those in the pore, i.e., by counting the molecules of H_2_O, CH_4_, H_2_ and CO_2_ in the two boxes at equilibrium. From top to bottom, the results are obtained with increasing pressure.

Results from Fig. 4 clearly show the pronounced effect of confinement on the reaction yield (bulk system adjacent to nanoporous matrix). While the bulk composition and the system total composition (left and middle panels) have somewhat similar trends with the reaction yields observed for the pure bulk systems discussed above (i.e., see Fig. 2), the pore composition is distinctively different, with water as the dominant substance in all cases. In the confined phase, only negligible amounts of CO_2_ and H_2_ are detectable, while CH_4_ can be adsorbed in a substantial amount. The behavior observed for the various fluid molecules is different due to a combination of effects, including the overall composition of the system, the preferential adsorption of water on the pore surfaces considered here, where water can form hydrogen bonds more readily than CO_2_, and to confinement-specific effects, which in some cases can strongly affect the solubility of methane in water^30^.

**Figure 5:**
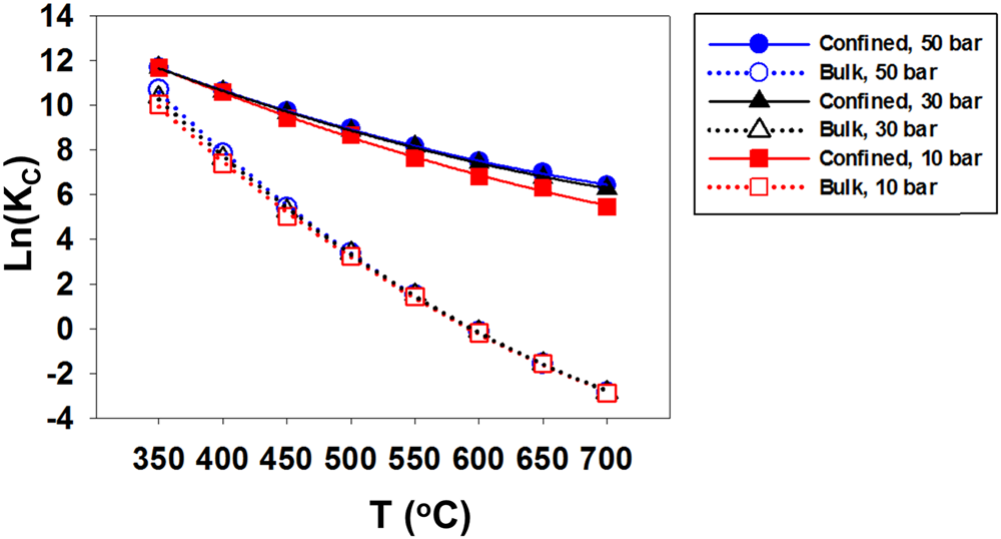
Predicted shift in concentration quotients *K*_*C*_ for pure bulk versus confined phases at *P* = 10, 30 and 50 bar.

**Figure 6:**
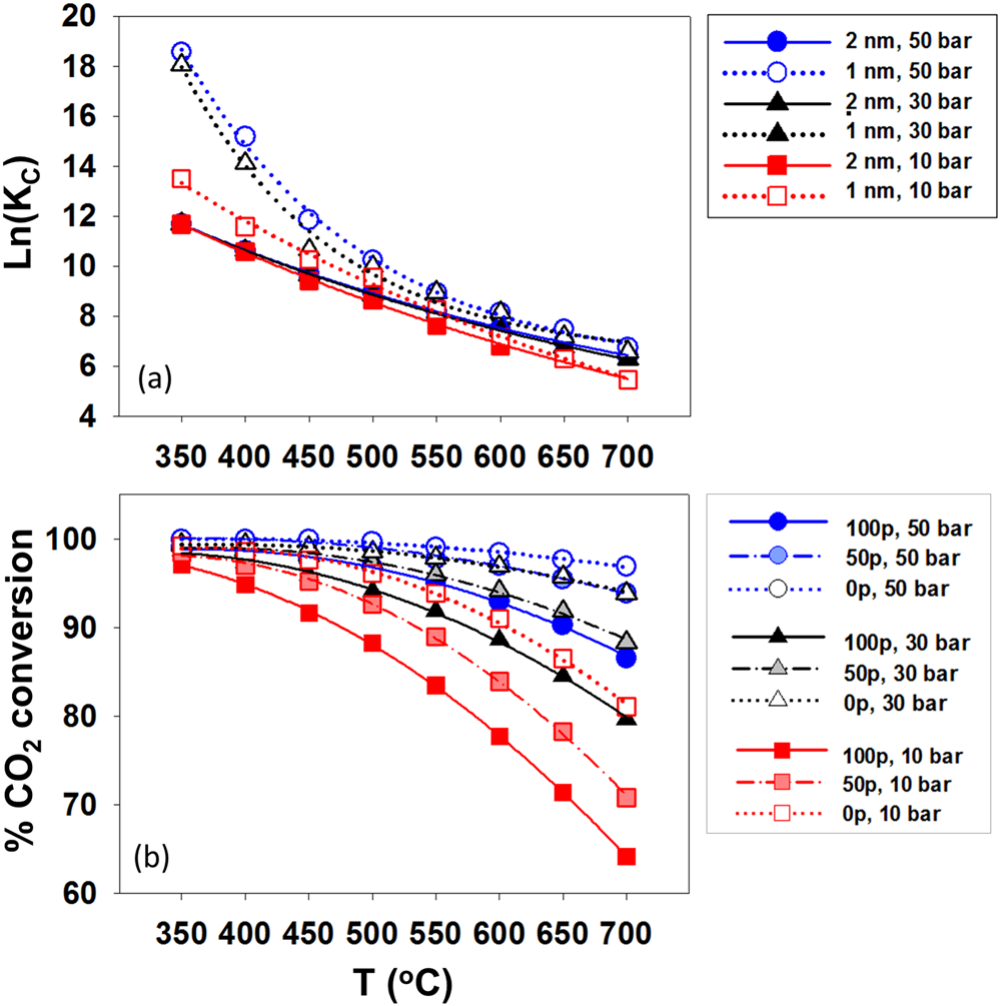
(**a**) Confined *K*_*C*_ when the reaction occurs for a system in contact with slit-shape silica pores of width 1 nm versus 2 nm. (**b**) *Overall* carbon dioxide conversion for the silica substrates with different degrees of protonation. The fully protonated silica pore surface is denoted as “100p”; surfaces obtained by removing ~50% and 100% of the H atoms form the surface –OH groups are denoted as “50p” and “0p”, respectively. Dotted, dashed and continuous lines in panel (**b**) are for results obtained at 10, 30, and 50 bar, respectively.

From the equilibrium mole fractions, we can calculate the shifts in the equilibrium composition of the system due to the effect of confinement on the equilibrium conversion as a function of *T* and *P*. Shifts in conversion can be assessed via concentration quotients, *K*_*C*_, computed by considering the composition of the whole system (i.e., middle panels in Fig. 4). Explicitly:

5 $${K}_{C}=\frac{{x}_{CH4}\times {x}_{H2O}^{2}}{{x}_{CO2}\times {x}_{H2}^{4}}$$

where *x*_*i*_ is the mole fraction of component i. Since the relative amount of confined vs. bulk phases is expected to affect the overall composition, in Fig. 5 we present *K*_*C*_ separately for pure bulk phase and confined phase as obtained using the mole fraction values shown from Fig. 2 and from the right panels of Fig. 4, respectively. It can be seen that the effect of temperature on the composition at equilibrium is much stronger than that of pressure for the range of *T* and *P* considered. Because the composition of the confined system is significantly different compared to the bulk at all pressures and temperatures considered, our results indicate that the *K*_*C*_ values calculated from the fluid composition in the pore can be very different than those obtained in the bulk. These observations are due to the fact that the presence of the silica pores effectively reduces the mole fraction of water, a reaction product, from the bulk phase. As a consequence, the equilibrium of Reaction (3) in the bulk phase is shifted further to the right, yielding a final CO_2_ conversion of the 2-phase system higher than that of the single bulk phase system. As shown in Fig. 3, water is preferentially adsorbed within the pores considered in this study. The driving forces responsible for this effect are discussed below (Fig. 6 and Supplementary Information). These results strengthen the arguments discussed by McDermott *et al*. regarding possible pathways for abiotic organic synthesis at submarine hydrothermal fields^10^. The results in Fig. 5 also suggest that increasing P as a modest, yet visible effect in increasing *K*_*C*_ for the confined system. This is likely because as P increases more water adsorbs within the pores considered here.

We note that the overall conversion of the chemical reaction can be shifted by changing the amount of pore volume in the host matrix with respect to the bulk volume, as controlling this ratio while maintaining pore width and pore chemistry allows manipulating the amount of water present in the bulk phase. For example, if the pore X and Y dimension increases while the bulk volume remains constant, more water would adsorb in the pore, lowering the water mole fraction in the bulk phase and shifting the equilibrium further towards CH_4_ production.

#### Effects of Pore Size, Pore Chemistry and Surface Roughness

In natural systems, pore and fracture networks can exhibit a wide range of pore size (or aperture width), pore volume and the pore surfaces may be highly heterogeneous, both in chemical composition and morphological structure. In general, there are more small fractures compared to large ones and pore and fracture networks show fractal relationships^74^. In this section, the effects of the pore size, pore chemistry and surface roughness on overall system equilibrium are quantified, as they are all expected to determine the preferential adsorption of water.

Figure 6 (panel a) compares *K*_*C*_ in confinement for simulations such as those discussed in Fig. 4 when the slit-shaped silica pore width is reduced from 2 nm to 1 nm. The reduction of the pore width results in two important outcomes. While the density of confined water increases (see Supplementary Information, S3) the pore volume is reduced by a factor of ~2. Recall that in our RxMC simulations the pore volume remains constant, while the bulk volume fluctuates to maintain the bulk *pressure* constant. Because the *total amount* of adsorbed water in the narrower pore is less than that in the 2 nm pore at higher *T* (~500–700 °C), the corresponding *X*_*CO2-1nm*_ is lower than *X*_*CO2-2nm*_ (panel a) in this range. At 300 and 500 °C the opposite is observed. Because the amount of water adsorbed in the pores considered here depends on many factors, including temperature, pressure, pore width, and, as described below, pore chemistry, the effects of these parameters on the equilibrium composition strongly depend on the system conditions. For example, as shown in Fig. 6, panel a, *K*_*C*_ obtained for the conditions chosen here can be orders of magnitude larger when the pore is 1 nm rather than 2 nm, at low P and T, but it becomes similar at high P and high T.

The effect of pore wall chemistry was quantified by varying the ‘hydrophilicity’ of the pore surface from 100% hydrated, to 50% and then to 0% (S4 in the Supplementary Information). While prior simulations were conducted using molecular dynamics^26^, our Monte Carlo approach yields consistent results, as shown in S4, wherein the number of water molecules adsorbed in the various pores increases with decreasing concentration of H surface atoms. In panel (b) of Fig. 6 we show the effect of water adsorption on CO_2_ conversion, which is quite pronounced. It should be pointed out that depending on the system pH the protonation state of the non-bridging oxygen atoms varies, with a strong effect on the conversion for the methanation reaction of CO_2_, as quantified by our calculations. In a variation on this theme, we *decreased* the pore hydrophilicity by covering the silica surface with a model carbon-based material – i.e., methane (see Supplementary Information S5–7). As the number of such carbon-based model molecules placed on the pore surface increases, the number of water molecules adsorbed decreases, hence reducing the reaction yield.

Finally, we explored the effect of the pore surface morphology/roughness by inserting two step-edges along the Y-direction^31^ (hence creating a structured pore). The step edges are chosen as examples of small defects on the pore surface. They are expected to render the pore surfaces more hydrophilic because more hydrogen bonds should be possible with water molecules. Our simulations confirm this expectation. A sample snapshot of the pore is shown in Supplementary Information, Figure S8. It can be seen that water out competes CO_2_ and accumulates near the surface edges (see Supplementary Information). When we compare the amount of water molecules adsorbed in the pores where the line defects are present against those with pristine surfaces (Figure S9), we observe that the defective pores adsorb more water, especially at low pressures. This is expected to affect the extent of the reaction. Results in terms of % CO_2_ converted (Figure S9) indeed show a slight increase in the pore whose surfaces contain step-edge defects. Although the differences in % CO_2_ converted may not be statistically significant, it should be remembered that step edges are just small defects. It is possible that larger effects would be obtained when more realistic, rougher surfaces are considered. Such analysis is beyond the scopes of the present work.

## Conclusions

In summary, reactive ensemble Monte Carlo simulations were conducted for the methanation reaction in either the bulk phase or for the bulk phase in equilibrium with a silica nanopore (a fracture-nanopore network system). In agreement with prior reports, the results in the bulk phase show that high pressures and low temperatures favour the production of methane from CO_2_. The situation can be affected by the preferential adsorption of fluids in nanopores. To explicitly consider the effect of confinement, we generated slit-shaped pores carved out of silica, constructed from β–cristobalite crystals. Indeed, preferential adsorption of the fluids in the narrow pores can affect the equilibrium composition. In agreement with Le Chatelier’s principle, when water, one product, is preferentially adsorbed, “*the position of equilibrium moves to partially reverse the change*”, yielding more water, and consequently more methane, thus enhancing the overall reaction conversion. The effects of pore size, pore chemistry and pore morphology were investigated. All conditions that enhance water adsorption (i.e., increasing pore hydrophilicity or roughness) result in an enhancement of reaction yield.

Our study could shed light on the possibility of thermal conversion of CO_2_ to hydrocarbons within hydrothermal vent systems. Should the Sabatier reaction occur in these environments, it would take place within the oceanic crust perhaps influenced by strong confinement effects. Confinement can shift the equilibrium composition towards facilitating methane formation at conditions that should not be strongly kinetically limited (i.e., moderately high temperature and low pressure). These results support the possible existence of realistic pathways for the abiotic organic synthesis discussed by, e.g., McDermott and coworkers^10^.

## Methods

### Reactive Ensemble Monte Carlo

Turner *et al*.^37, 38^ reported the details concerning the RxMC approach combined with the Gibbs ensemble MC algorithm (GEMC). The RxMC approach is designed to determine the equilibrium conditions (by minimizing Gibbs energy at constant temperature and pressure), regardless of the reaction rate and mechanism. To implement the method it is necessary to quantify the probabilities of the forward, *P*_*f*_, and reverse, *P*_*r*_, reactions, which, for Reaction (3), are:

6 $${P}_{f}=\,\min \{1,(\frac{{q}_{C{H}_{4}}{q}_{{H}_{2}{O}^{2}}}{{q}_{C{O}_{2}}{q}_{{{H}_{2}}^{4}}})[\frac{({N}_{C{O}_{2}})({N}_{{H}_{2}})({N}_{{H}_{2}}-1)({N}_{{H}_{2}}-2)({N}_{{H}_{2}}-3)}{({N}_{C{H}_{4}}+1)({N}_{{H}_{2}O}+1)({N}_{{H}_{2}O}+2)}]\exp (-\beta {\Delta }{U}_{f})\}$$

7 $${P}_{r}=\,\min \{1,(\frac{{q}_{C{O}_{2}}{q}_{{{H}_{2}}^{4}}}{{q}_{C{H}_{4}}{q}_{{H}_{2}{O}^{2}}})[\frac{({N}_{C{H}_{4}})({N}_{{H}_{2}O})({N}_{{H}_{2}O}-1)}{({N}_{C{O}_{2}}+1)({N}_{{H}_{2}}+1)({N}_{{H}_{2}}+2)({N}_{{H}_{2}}+3)({N}_{{H}_{2}}+4)}]\exp (-\beta {\Delta }{U}_{r})\}$$

In equations (6) and (7) *q*_*i*_ is the partition function of specie *i*; $\Delta {U}_{f}$ and $\Delta {U}_{r}$ are changes in the system configurational energy for the forward and reverse reactions, respectively; *N* is the number of molecules and *β* = 1/(*k*_B_ *T*) where *k*_B_ is the Boltzmann constant.

Our simulations contain the following trial moves:

1. Particle displacement/rotation step.
2. Forward reaction step:
  - Delete one CO_2_ and four H_2_ molecules randomly;
  - Insert one CH_4_ and two H_2_O molecules with random orientation;
  - Accept the move with probability ${P}_{f}$.
3. Reverse reaction step:
  - Delete one CH_4_ and two H_2_O molecules randomly;
  - Insert one CO_2_ and four H_2_ molecules with random orientation;
  - Accept the move with probability ${P}_{r}$.
4. Phase exchange step for a random particle with probability $\,{P}^{t}$.
5. Change in bulk volume with probability $\,{P}^{v}$.

It is crucial that steps (2) and (3) are chosen with equal probability to maintain microscopic reversibility. Steps (1), (2), (3) and (4) are selected 50, 20, 20, and 10% of the time, respectively, following prior implementations^37^.

For all simulations, an initial composition of 1000 CO_2_ and H_2_ molecules was used, with a molar ratio 1:4. While this molar ratio is chosen to better visualise the effect of confinement on the equilibrium of Reaction (3), it should be noted that analytical data from representative hydrothermal vents suggest that the CO_2_:H_2_ molar ratio in those environments can be as high as 10 or more, but in a few cases the ratio can be lower than 1^4^. All systems were allowed to equilibrate for 1 × 10^6^ moves and the averages were analyzed for 2 × 10^6^ moves. Reaction equilibrium was satisfied by the following criteria:

8 $$\sum _{i=1}^{s}{v}_{i}{\mu }_{i}=0$$

where ${v}_{i}$ and ${\mu }_{i}$ denote the stoichiometric coefficient and chemical potential of species *i* for the mixture of *S* components, respectively, as calculated during the RxMC algorithm.

### Molecular Models

Hydrogen and methane molecules were treated as monoatomic spheres, while each water and carbon dioxide molecule had three Lennard-Jones (LJ) sites and three charge sites. Water was simulated using the SPC/E model^75^ while CO_2_ and CH_4_ were simulated using the TraPPE-UA force field^76^. LJ interaction parameters for H_2_ were taken from Huber and Herzberg^77^. Dispersive and electrostatic interactions were modelled with the 12–6 LJ and Coulombic potentials, respectively. LJ parameters (*ε* and *σ*) for non-like components obtained using Lorentz-Berthelot mixing rules [*ε*_*ij*_ = (*ε*_*i*_ *ε*_*j*_)^1/2^, *σ*_*ij*_ = (*σ*_*i*_ + *σ*_*j*_)/2]^78^. The cut off distance for both LJ and electrostatic interactions was set at 1.4 nm, following TraPPE-UA prescriptions^76^. Long-range corrections include (1) tail correction method for LJ potential and pressure calculation and (2) Onsager reaction field method for electrostatic interactions. All molecular models are simplifications. The TraPPE force fields were parameterised to reproduce vapor-liquid equilibria experimental data. The SPC/E model for water was introduced to improve the ability of the single point charge model (SPC) to reproduce the structure of liquid water. This was achieved by increasing the dipole moment of the individual water molecule, with the result that the predicted saturation pressure is much lower than experimental observations, and that water-water interactions in the vapour phase are much more attractive for the SPC/E model than they are in reality. Given the high temperature of the systems considered here, this limitation is not expected to affect the results presented. None of the models used here allows us to describe molecular dissociations, nor associations. We are implementing reactive force fields for such analysis. The results presented are dependent on the ability of the models to describe the preferential adsorption of the various fluids in the pores, and also on the interactions between molecules of different fluids. Based on prior observations, we expect that the models implemented here describe water-silica interactions rather accurately.

Vibrational frequencies and rotational constants were obtained from the literature^79–81^. The dissociation energy *D*_0_, used to calculate the partition function *q*_*i*_ from its ground-state value, can be determined from the heat of formation at 0 K. For a specific molecule, the molecular *D*_0_ equals the sum of Δ_*f*_ *H* ^0^ from all its individual atoms, subtracting Δ_*f*_ *H* ^0^ of the molecule itself.

### Silica Slit-Shaped Pore Model

A detailed description of the solid morphology used to model the solid substrate is provided elsewhere^25, 28^. The CLAYFF force field is used to describe the solid substrate, as discussed elsewhere^81^. The solid substrates are maintained rigid during these simulations. Because of periodic boundary conditions, the systems considered are composed by silica slabs that are infinitely long along the X and Y directions, and separated along the Z direction by the slit-shaped pore. In our model the solid substrate bears no net charge, and all the non-bridging O atoms at the pore surface are fully protonated, yielding a density of surface –OH groups equal to 13.6 OH/nm^2^.

### Data Availability Statement

The authors declare that all data supporting the findings of this study (either calculated or cited from external sources) are available within the article and its supplementary information files.

## Electronic supplementary material

Confinement Effects on Carbon Dioxide Methanation: A Novel Mechanism for Abiotic Methane Formation
