# Supplementary material for: Confinement Effects on Carbon Dioxide Methanation: A Novel Mechanism for Abiotic Methane Formation
Source: Sci Rep. 2017 Aug 21;7:9021. doi: 10.1038/s41598-017-09445-1 (PMC5566444; doi:10.1038/s41598-017-09445-1)
Supplement: Supplementary file 1 — Confinement Effects on Carbon Dioxide Methanation: A Novel Mechanism for Abiotic Methane Formation [file 41598_2017_9445_MOESM1_ESM.doc]

**Confinement Effects on Carbon Dioxide Methanation:**

**A Novel Mechanism for Abiotic Methane Formation**

Thu Le and Alberto Striolo*

*Department of Chemical Engineering, University College London, London WC1E 6BT
United Kingdom*

C. Heath Turner

*Department of Chemical and Biological Engineering, University of Alabama, Tuscaloosa, AL 35487
United States*

David R. Cole

*School of Earth Sciences, The Ohio State University, Columbus, Ohio 43210*

*United States*

**Supplementary Information**

**S1-2 Simulation of bulk fluid behaviour.**

In **Figure S1** we report the equilibrium constant. In all simulations, bulk systems are considered with *P* =1.01 bar and CO_2_:H_2_ input ratio 1:4.


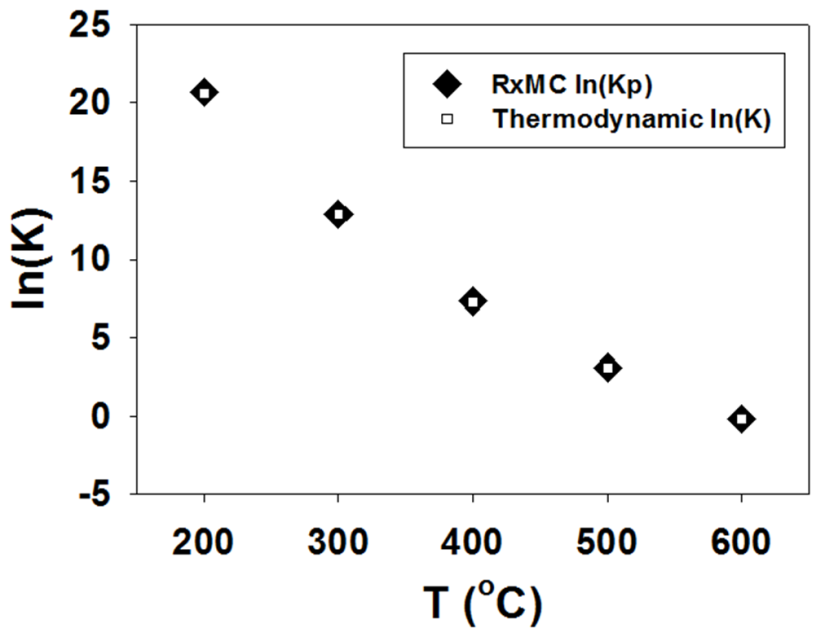


**Figure S1.** Simulated equilibrium constant compared to thermodynamic calculations as a function of temperature for the CO_2_ methanation reaction, Reaction [3].

Results in **Figure S1** demonstrate excellent agreement between the equilibrium constants obtained from classical thermodynamics[^1^](#_ENREF_1) versus those obtained by simulations implementing the RxMC algorithm. For completeness we point out that to better match the thermodynamic results the total atomization energy *D*_0_ was slightly adjusted (-150.1 kJ/mol instead of -151.29 kJ/mol)[^2^](#_ENREF_2). We estimate that varying *D*_0_ by 1.09 kJ/mol changes the equilibrium constant ln(
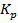
) by 6.9% for the cases considered in **Figure S1**. The conclusions of the present study do not depend on the choice of this parameter, as the value -150.1 kJ/mol was maintained constant for all subsequent simulations discussed in this manuscript.

It is important to point out that the *absolute*
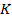
 is related to
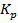
 by
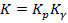
 with
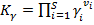
, where
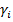
 is the activity coefficient of component *i*, and the product includes all *S* components in the system. Because
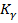
 is not equal to unity, there can be a difference of up to 7.3% at 200°C, between absolute *K* and *K_p_* for the system considered in **Figure S1**.

**
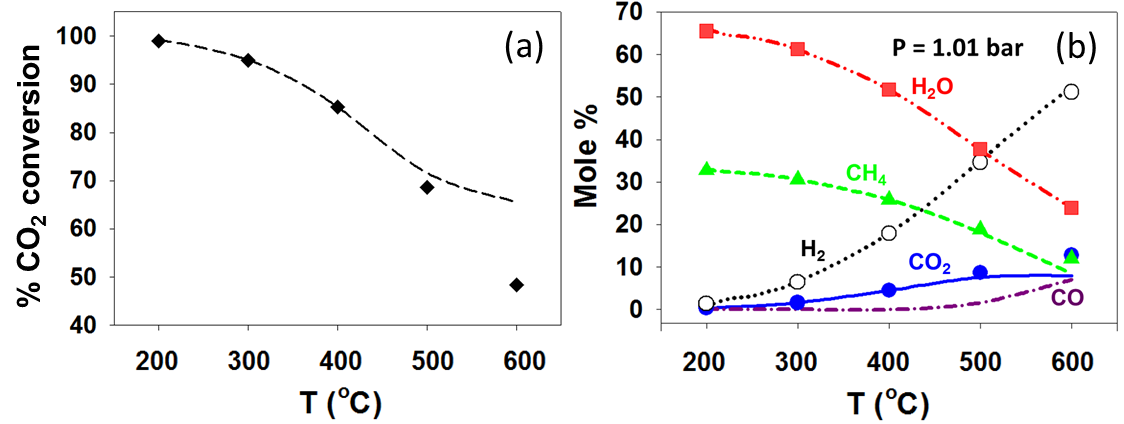
**

**Figure S2.** (a) Carbon dioxide conversion to CH_4_ and (b) mole percentage of all compounds involved in the CO_2_ methanation at equilibrium obtained by Gibbs free energy minimization (lines) [^3^](#_ENREF_3) versus simulations (symbols). The error bars are smaller than the symbols. Note that CO was not simulated, hence only literature results (dash-dot line) are shown for this compound.

Panel (a) of **Figure S2** compares CO_2_ conversion calculated by Gibbs free energy minimization by Gao et al.[^3^](#_ENREF_3), versus RxMC results calculated here as a function of temperature^63^. Good agreement is obtained at low *T* but the results are markedly different as *T* increases. Panel (b) shows that the molar fractions at equilibrium obtained from the two methods are almost identical: CH_4_ production is highly favourable at low *T* but its mole fraction decreases as *T* increases. At high temperature the simulated CO_2_ conversion and CH_4_ mole fraction predicted by the RxMC method are higher than the values reported by Gao et al.[^3^](#_ENREF_3) This is a consequence of the fact that Gao et al. considered **Reaction [4]**, with the formation of a CO intermediate, whereas we consider **Reaction [3]** with no CO allowed to form. With the two-step mechanism considered by Gao et al., some of the CO intermediate will react with water to form CO_2_ while the remainder reacts with H_2_ to form CH_4_. At low *T* the production of CO is so small in Reaction [4] that the two approaches yield undistinguishable results. Despite such differences, our results suggest that the RxMC approach yields the expected conversions when implemented in the bulk.

**S3. Effect of pore size**

**
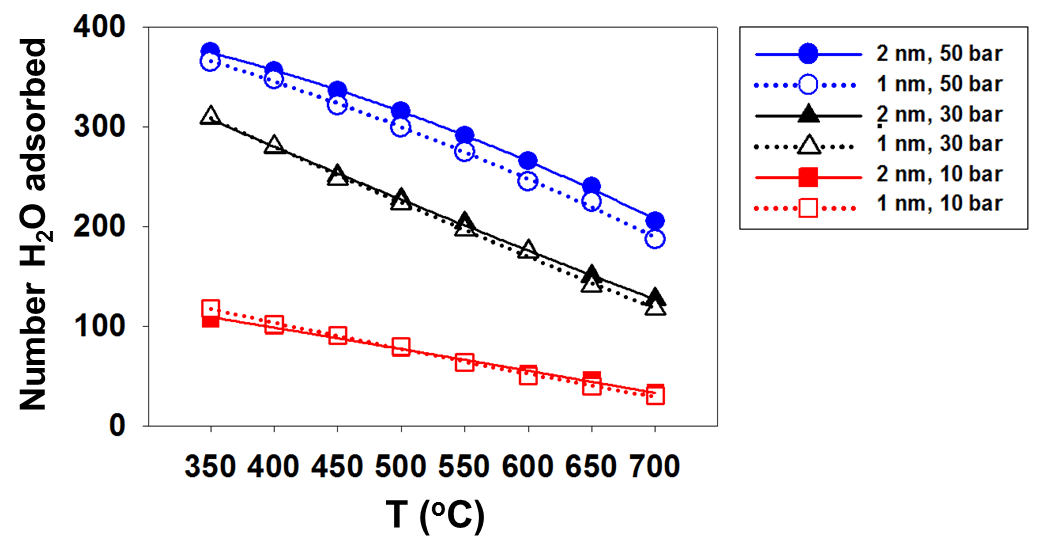
**


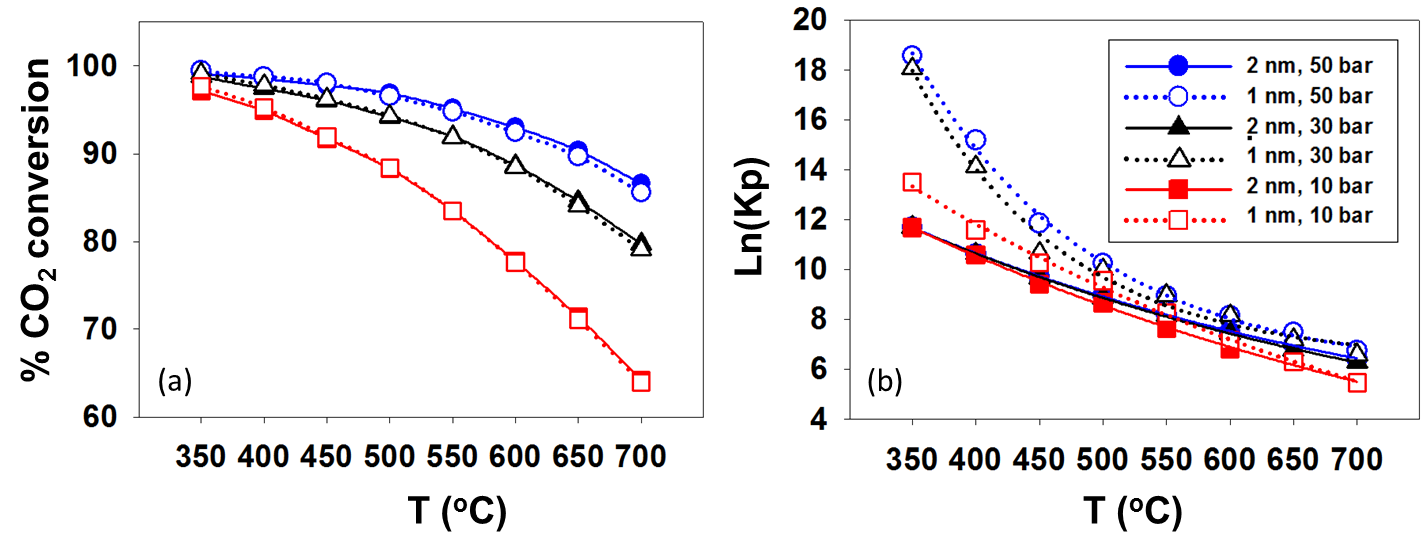


**Figure S3.** Top: Comparison for the number of water adsorbed in the confined phase within silica nanopore of 2 nm versus 1 nm width. Bottom: Carbon dioxide conversion to CH_4_ for a system in contact with slit-shape silica pores of width 1 nm versus 2 nm.

**S4. Effect of hydrophilicity on fluid behaviour in nanopores**


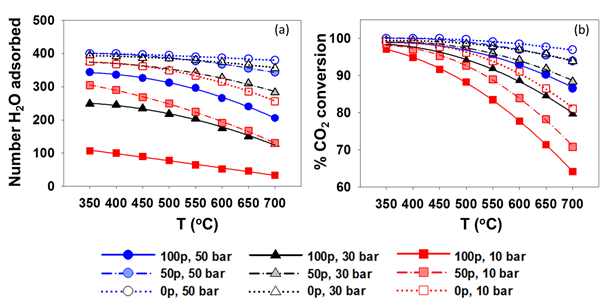


**Figure S4.** (a) Number of water adsorbed in the pore phase and (b) *overall* carbon dioxide conversion for the silica substrates with different degrees of protonation. The fully protonated silica pore surface is denoted as “100p”; surfaces obtained by removing ~50% and 100% of the H atoms form the surface –OH groups are denoted as “50p” and “0p”, respectively.

**S5-7. Model hydrophobic pore surfaces**

Canonical ensemble molecular dynamics simulation was run for 3000 methane molecules confined in the silica pore of 2 nm at 500°C. The density profile of methane across the pore width was produced and shown in **Figure S5**. Methane molecules that did not belong to the first adsorbed layers were removed. Those methane molecules on the pore surface were then considered part of the pore surface, and treated as rigid in subsequent simulations. The final configurations had either (1) 1132 methane molecules lying within a distance of *D* = 3.74 Å (which roughly equals the methane diameter of 3.73 Å)[^4^](#_ENREF_4) or (2) 420 methane lying within a distance of *D*_½_ = 1.87 Å away from the silica pore walls (see **Figure S5** for illustration). Since methane molecules bear no charge, no charge adjustment are required for the silica pore. Simulated snapshots and contour plots for methane planar densities on top silica surface for the two cases are shown in **Figure S6**.


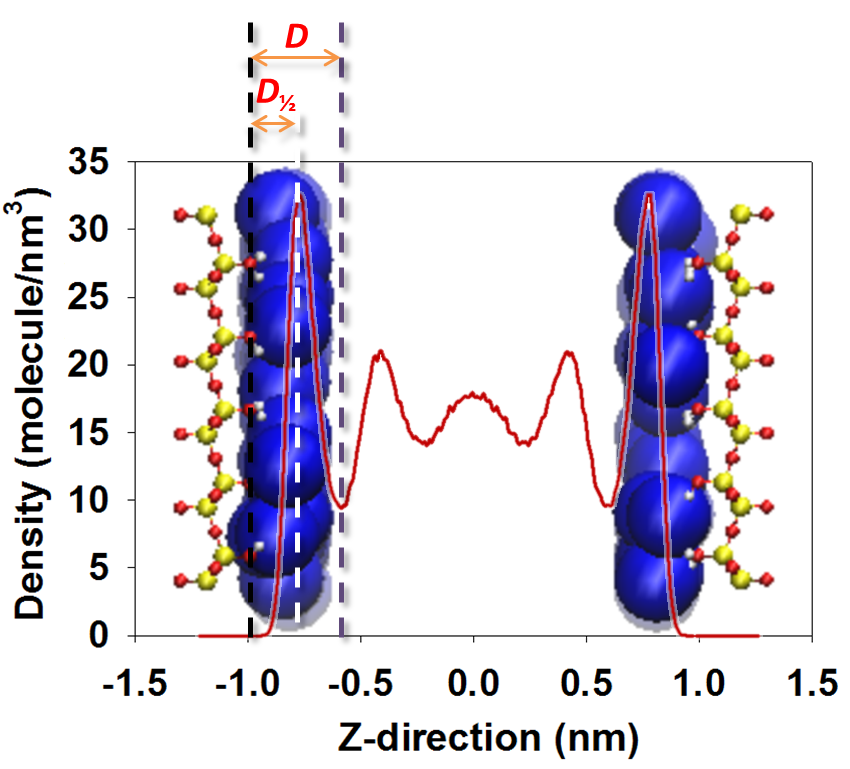


**Figure** **S5.** Molecular density profile of 3000 methane confined in 2 nm silica pore at 500°C and illustration for the methane molecules chosen for coating purpose. Blue spheres are the adsorbed CH_4_, red is O, white is H, and yellow is Si.


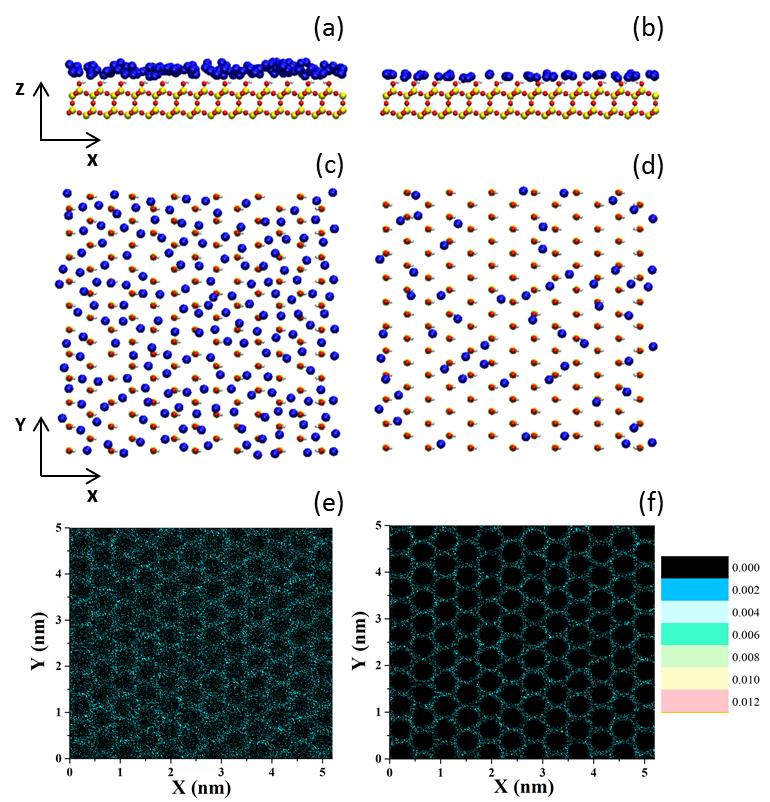


**Figure S6** (a), (b), (c), (d) Simulated snapshots of the first adsorbed layers of 1132 (left panels) and 420 (right panels) methane molecules on silica surface along different planes, respectively. Only a few layers of silica are shown for clarity. Colour scheme is the same as that of **Figure S5**. (e), (f) Corresponding contour plots of methane planar densities along X-Y plane. Densities are expressed in number of molecular COM per 10^-4^nm^2^.


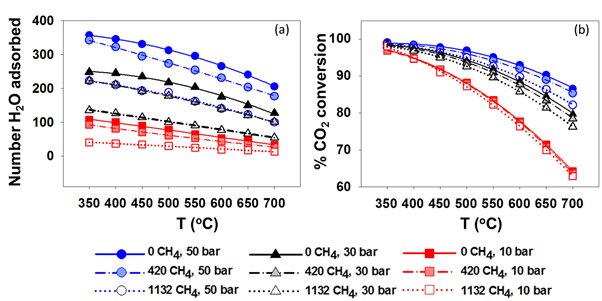


**Figure S7.** (a) Number of water adsorbed in the pore phase and (b) *overall* carbon dioxide conversion when the silica substrates are covered with different numbers of methane molecules. Note that the pristine silica pore surface is denoted as “0 CH_4_”.

**S8--9. Structured pore with a trench along the Y direction**

**
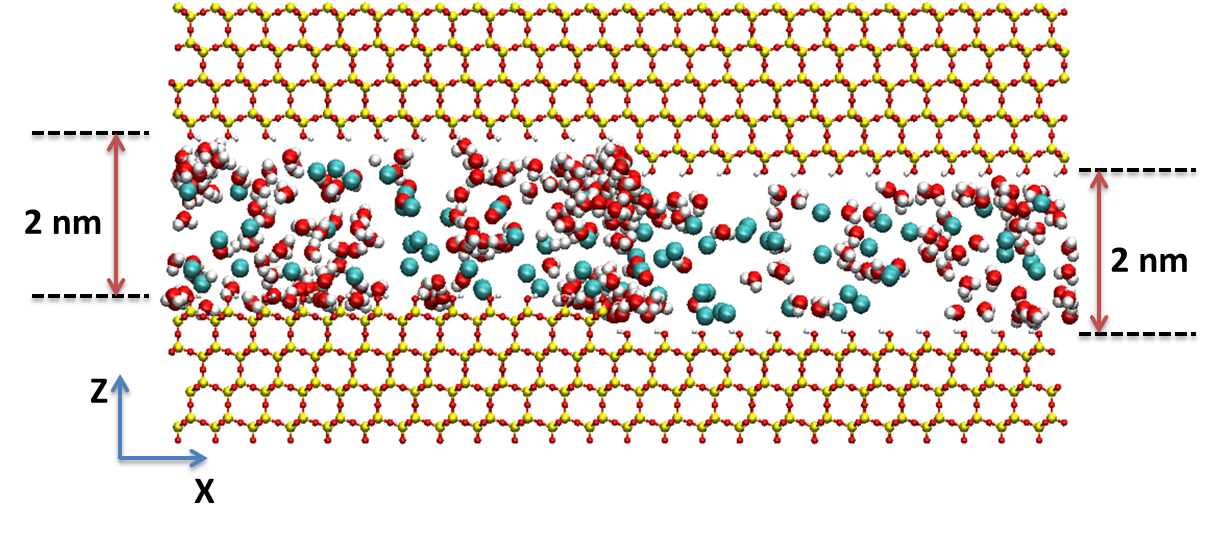
**

**Figure S8.** Simulated snapshot representing a simulation box containing the 2 nm structured silica pore at 650°C and 50 bar. The solid silica slabs are continuous along both X and Y directions. Cyan spheres are either CH_4_ or C in carbon dioxide, red is O, white is either H or H_2_, and yellow is Si.


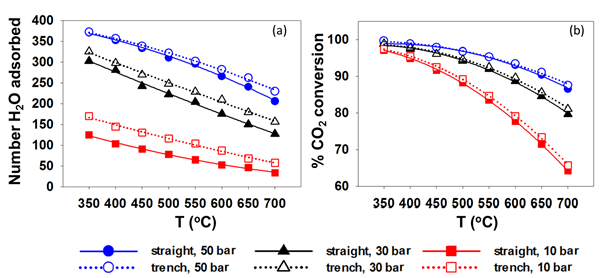


**Figure S9.** (a) Number of water adsorbed in the pore phase and (b) *overall* carbon dioxide conversion for silica substrates of different morphologies. In the figure legend, the term “straight” refers to the pristine silica pore (see **Figure 5a**) while “trench” refers to the structured pore (see **Figure S4**).

**References**
